# Malpractice in Vermont

**Published:** 1856-06

**Authors:** 


*Malpractice in Vermont.*—Dr. W. H. Thayer, the talented Professor of Theory and Practice, and Pathology, at the Woodstock school, has sent us a *resumé* of a malpractice suit which was recently decided at that place. The case was one which strikingly illustrated the peculiar beauties of that sort of thing, and the generous high principled course of action which prosecuting parties generally pursue. The *modus operandi*, though rather common in New England, is, we believe, peculiar to that section of the country. The "dodge" consists in the calling in by the noble-minded sufferer, for a momentary inspection of his fracture, some old practitioner, who, by the long years of his patient toil, has accumulated more or less of a competency. Having thus so to speak effected an insurance, he waits in hope of being blessed by a deformity. An airing of a dozen or two miles over a Vermont road, in a lumber-wagon, as in the case before us, or a prudent loosening of bandages and removal of splints, made during the absence, and without the knowledge, of the regular attendant, have been found to considerably increase the chances of the desired issue. Then, after a comfortable deformity is well established, the unfortunate who has only had a five-minute view of the case, is called on to support the poor victim in dignified ease during the rest of his natural life. Unfortunately, however, juries in that part of the world are getting to understand how the thing is done, and with a hardness of heart uncommon to jurymen, they are commencing to refuse to the poor sufferer the desired consolation, and it really would seem as if the claim of physicians to equal rights with other men, was, in New England at least, receiving some consideration.

Dr. Thayer has related the case of Closson vs. Loomis, in a succinct, straightforward, fair way, and a similar exposure should be made of every case of malpractice which occurs, to which general circulation should be given. The iniquity and injustice of these cases is never more apparent than when they are fairly reported by an honest, able-minded man.

